# Biogeographic and Habitat-Associated Variation in the Gut Microbiota of the Stingless Bee *Tetragonula laeviceps* Between Bali and Lombok, Two Islands Situated on Opposite Sides of the Wallace Line

**DOI:** 10.3390/insects17070733

**Published:** 2026-07-16

**Authors:** Joko Pilianto, Amr Abou El-Ela, Jinxing Li, Tianxiang Du, Mochammad Syamsul Hadi, Asim Munawar, Kamila Muyasarah, Naved A. Ansari, Wenwu Zhou, Zengrong Zhu, Dun Wang

**Affiliations:** 1 College of Plant Protection, Northwest A&F University, Yangling 712100, China; joko.pilianto@gmail.com (J.P.); ljxcykc@163.com (J.L.); dutxiang@nwsuaf.edu.cn (T.D.); 2 Department of Plant Pests and Disease, University of Brawijaya, Malang 65145, Indonesia; msh@ub.ac.id; 3 State Key Laboratory of Rice Biology and Breeding, Ministry of Agriculture and Rural Affairs Key Laboratory of Molecular Biology of Crop Pathogens and Insect Pests, Institute of Insect Sciences, Zhejiang University, Hangzhou 310058, China; dr_amraboelela@alexu.edu.eg (A.A.E.-E.); navedahmad.ansari@gmail.com (N.A.A.); .; zrzhu@zju.edu.cn (Z.Z.); 4 Plant Protection Department, Faculty of Agriculture (Saba Basha), Alexandria University, Alexandria 21531, Egypt; 5 Harvard Medical School, Harvard University, Boston, MA 02115, USA; kamila_muyasarah@hms.harvard.edu

**Keywords:** stingless bee, 16S rRNA sequencing, microbiome, biogeography, forest habitat, agroforestry, tropical pollinators, Indonesia

## Abstract

Biogeographic barriers are well known to structure animal and plant diversity, but whether they are associated with variation in host-associated microbiomes, especially in tropical pollinators, remains poorly understood. Here, we investigated gut bacterial community variation in the stingless bee *Tetragonula laeviceps* (Apidae: Meliponini) collected from Bali and Lombok, two Indonesian islands situated on opposite sides of the Wallace Line. Using 16S rRNA gene V3–V4 amplicon sequencing, we analyzed 12 colony-level samples collected from forest and agroforestry habitats on both islands. Alpha-diversity analysis revealed variation in bacterial richness and diversity among colonies and sampling groups, with the highest richness observed in the Lombok agroforestry group. The gut microbiota was dominated by *Firmicutes*, *Proteobacteria*, *Actinobacteriota* and *Bacteroidota*, with several bee-associated genera, including *Lactobacillus*, *Bombilactobacillus*, *Apilactobacillus*, *Bombella*, *Commensalibacter*, *Snodgrassella*, *Bifidobacterium* and *Bartonella*, varying in relative abundance among island and habitat groups. Beta-diversity analyses showed significant group-associated differentiation in bacterial community composition (ANOSIM *R* = 0.444, *p* = 0.001), indicating that gut microbiome structure differed across the sampled island–habitat groups. Differential-abundance analyses identified candidate bacterial taxa associated with specific groups, suggesting that microbiome divergence was driven mainly by shifts in the relative abundance of shared bacterial lineages rather than complete replacement of dominant taxa. Predicted functional profiling further indicated that the taxonomic variation was accompanied by differences in putative functional potential. These findings suggest that the gut microbiota of *T. laeviceps* carries a detectable island and habitat-associated signal and highlight host-associated microbial communities as an underexplored dimension of tropical island biogeography. Because island identity and position relative to the Wallace Line are not independent in this design, these results should be interpreted as island associated microbiome variation within a Wallace Line context, not as direct evidence of a causal Wallace Line effect.

## Simple Summary

Stingless bees, *Tetragonula laeviceps* are important pollinators in tropical ecosystems, but little is known about how their gut bacteria differ between islands and habitats. In this study, we examined bees from two Indonesian islands, Bali and Lombok, collected from both forests and agricultural areas. We found that the types and amounts of gut bacteria varied depending on the island and habitat. Some bacterial groups were more common in one island or habitat than another, while others were shared across all samples. These differences were not due to entirely new bacteria appearing, but rather changes in the abundance of existing bacteria. Our analyses also suggested that the gut bacteria could perform different functions, such as helping with digestion, metabolism and other activities important for bee health. Furthermore, the interactions among bacterial groups showed a connected but flexible network rather than being dominated by a few types. Because this study included only Bali and Lombok, the Wallace Line is interpreted as a biogeographic context rather than as an independent causal factor. Overall, these findings reveal that the gut microbiome of *T. laeviceps* reflects the environment in which they live, including both the island and habitat type.

## 1. Introduction

Biogeographic barriers play a central role in shaping the distribution, diversification and evolutionary trajectories of organisms across the globe [1,2]. Among the most iconic of these boundaries is the Wallace Line, first proposed by Alfred Russel Wallace during the nineteenth century to explain the abrupt faunal transition between the Asian and Australasian biotas across the Indonesian archipelago [3]. Despite the relatively short geographic distance separating Bali and Lombok, the deep Lombok Strait has persisted as a marine barrier through historical sea-level fluctuations, preventing direct land connection between Bali on the Sunda Shelf and Lombok within Wallacea. As a consequence, the Wallace Line has long been associated with pronounced shifts in vertebrate, invertebrate and plant communities, as well as phylo-geographic divergence among populations distributed on opposite sides of this boundary [4,5,6]. Such host-population divergence may also influence host-associated microbial communities, because differences in host genetic background, physiology or microbial filtering can contribute to gut microbiota variation. Although the macro-ecological and evolutionary significance of the Wallace Line is well established, whether this biogeographic transition is also reflected in host-associated microbial communities, particularly those of tropical pollinators, remains poorly understood.

In recent years, host-associated microbiomes have emerged as critical components of animal biology, contributing to nutrition, immunity, development, detoxification, behavior and ecological adaptations [7]. The concept of the holobiont recognizes that hosts and their associated microorganisms function as integrated biological units whose interactions may influence ecological fitness and evolutionary processes [8]. Increasing evidence suggests that microbial communities are not randomly assembled but are shaped by a combination of host phylogeny, environmental conditions, geography, diet, habitat and social interactions [9,10,11]. Consequently, biogeographic barriers that structure macro-organismal diversity may also influence microbiome composition indirectly through variation in environmental microbial pools, floral resources, host population structure, nesting environments and ecological interactions [12,13,14]. However, compared with vertebrates and model insects, the biogeography of microbiomes in tropical pollinators remains insufficiently explored. Social bees harbor some of the best-characterized insect gut microbiomes and provide valuable systems for investigating host–microbe interactions [15,16,17]. Studies of honey bees and bumble bees have revealed relatively conserved gut bacterial communities that contribute to carbohydrate metabolism, pollen digestion, fermentation, pathogen defense, immune modulation and detoxification of dietary compounds [18,19]. Although social bee gut microbiota can show conserved host-associated structure, increasing evidence indicates that community composition may vary according to geography, environmental conditions, diet, floral resources, agricultural exposure and host species [15,16,20]. Such variation may influence pollinator health, resilience and adaptations to changing ecosystems.

Stingless bees (Apidae: Meliponini) are among the most ecologically important eusocial pollinators in tropical and subtropical ecosystems. More than 600 stingless bee species have been described worldwide, with high diversity in tropical regions, including Southeast Asia and the Neotropics [21,22]. In Indonesia, stingless bees play important ecological and economic roles through pollination services, honey and propolis production and meliponiculture [23,24]. Compared with honey bees and bumble bees, however, stingless bees’ microbiomes remain comparatively understudied, particularly in island biogeographic systems. Existing studies suggest that stingless bee gut microbiota includes bacterial lineages commonly associated with corbiculate bees, while also containing distinctive assemblages enriched in *Lactobacillaceae*, *Acetobacteraceae*, *Bifidobacteriaceae* and related bee-associated taxa [25,26]. These microbial communities are likely shaped by social transmission, nest environment, floral resources and local environmental conditions, but the extent to which large-scale biogeographic transitions contribute to microbiome divergence remains unclear [27]. *Tetragonula laeviceps* (Smith, 1857) is a widely distributed stingless bee species in Southeast Asia and is commonly recorded in Indonesia, making it a suitable model for investigating gut microbiome variation across island and habitat contexts [24,28]. Colonies occur in both Bali and Lombok under broadly comparable tropical climatic conditions, but within distinct biogeographic regions separated by the Lombok Strait. This geographic configuration provides a natural comparative framework for evaluating whether gut bacterial community structure differs between populations located on opposite sides of the Wallace Line, while also allowing habitat-associated variation to be assessed within each island [29]. Understanding such patterns is important not only for microbial ecology and pollinator biology, but also for broader questions concerning how biogeographic history and contemporary habitat conditions interact to shape host-associated microbial communities.

The present study investigated the gut bacterial communities of *T. laeviceps* colonies collected from Bali and Lombok, representing opposite sides of the Wallace Line. We further compared colonies from forest and agroforestry habitats within each island to assess island-associated and habitat-associated variation in microbiome structure. We hypothesized that: (I) *T. laeviceps* colonies from Bali and Lombok would differ in gut bacterial community composition, consistent with island-associated microbiome structuring between Bali and Lombok, two islands situated on opposite sides of the Wallace Line; and (II) habitat differences between forest and agroforestry systems would be associated with secondary shifts in bacterial composition and predicted functional profiles. This study provides insight into the relationship between island biogeography and pollinator associated microbiomes and contributes to a growing understanding of the microbial dimensions of tropical biodiversity.

## 2. Materials and Methods

### 2.1. Bee Sampling and Study Sites

**Figure 1:**
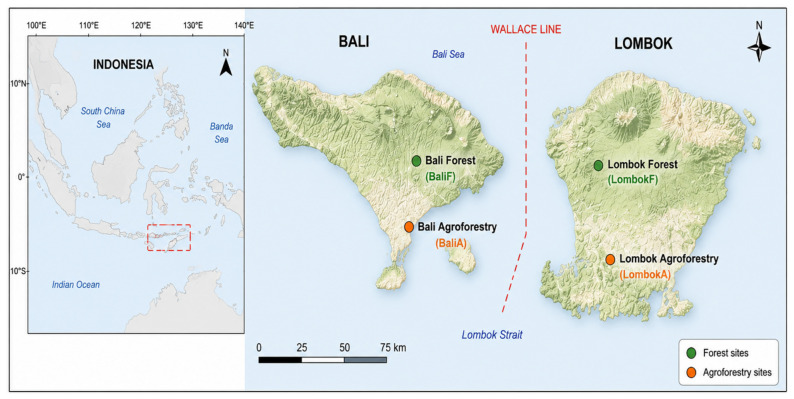
Study area and sampling locations across the Wallace Line. The dashed red line indicates the approximate position of the Wallace Line between Bali and Lombok. Green circles indicate forest sites and orange circles indicate agroforestry sites. The map was prepared using geographic base layers and topographic elements in QGIS v3.x, then refined for scientific visualization in Adobe Illustrator v. 30.6 (Adobe Inc., San Jose, CA, USA).

This study investigated the gut microbiota of the stingless bee *Tetragonula laeviceps* collected from Bali (approximately 8° S, 115° E; Sundaland, west of the Wallace Line) and Lombok (approximately 8.5° S, 116° E; Wallacea, east of the Wallace Line) Islands, Indonesia (Figure 1). Field sampling was conducted in November 2025, corresponding to the early wet season in Bali and Lombok. Sampling was designed to evaluate island-associated and habitat-associated variation in bacterial community composition.

Colonies were sampled from two habitat categories on each island: forest habitats (F) and agroforestry habitats (A), resulting in four sampling groups: Bali Forest (BaliF), Bali agroforestry (BaliA), Lombok Forest (LombokF) and Lombok agroforestry (LombokA). A total of 12 colonies were included in the study, with three independent colony-level biological replicates per sampling group. On each island, three colonies were sampled from primary or secondary forest habitats and three colonies from mixed-tree agroforestry systems dominated by coconut (*Cocos nucifera*), coffee (*Coffea* spp.) and assorted fruit trees.

Colony-level GPS coordinates, elevation, inter-colony distances, pesticide history, and detailed flowering-plant inventories were not fully recorded during sampling. Therefore, these unmeasured local environmental variables are acknowledged as potential contributors to microbiome variation. For each colony, *approximately* 30 returning adult worker foragers were collected at the nest entrance between 07:00 and 11:00 local time using sterile forceps; queens and drones were not included. Bees were immediately surface-sterilized with 70% ethanol, transferred to RNAlater-equivalent preservation buffer, and stored at −20 °C until gut dissection.

For downstream sequencing, guts from 30 worker foragers per colony were pooled to generate one colony-level gut sample. Therefore, each island × habitat group contained three pooled colony-level samples, corresponding to 90 worker guts per group and the full dataset contained 12.

### 2.2. DNA Extraction, Amplification and Sequencing

Gut tissues were dissected from worker bees on ice under a stereomicroscope (ZEISS Stereo Discovery V8) using sterile forceps and sterile scalpel blades under aseptic conditions. The small sample size limited statistical power, especially for alpha-diversity comparisons, differential abundance, predicted functions, and network analysis. For each colony, dissected guts from worker bees were pooled to generate one colony-level sample, immediately transferred into sterile micro-centrifuge tubes (Axygen) and stored at −80 °C until DNA extraction. Total microbial genomic DNA was extracted from gut samples using the Easy Tissue Genomic DNA Extraction Kit according to the manufacturer’s protocol. DNA concentration and purity were assessed using spectrophotometric analysis and agarose-gel electrophoresis prior to PCR amplification. The bacterial 16S rRNA gene V3–V4 region was amplified using the primer pair reported for the sequencing run: forward primer ACTCCTACGGGAGGCAGCA and reverse primer GGACTACHVGGGTWTCTAAT. The 16S rRNA V3–V4 region was amplified in a 25 μL PCR reaction containing 2.5 μL template DNA, 5 μL forward primer, 5 μL reverse primer and 12.5 μL 2× high-fidelity PCR master mix. PCR cycling consisted of initial denaturation at 95 °C for 3 min, followed by 25 cycles of 95 °C for 30 s, 55 °C for 30 s and 72 °C for 30 s, with a final extension at 72 °C for 5 min. Amplicons were purified using magnetic bead-based purification, indexed by limited-cycle PCR, pooled in equimolar concentrations and sequenced on the Illumina NovaSeq 6000 platform using paired-end [250/300]-bp reads. PCR amplification was performed under optimized cycling conditions and amplification products were verified using agarose-gel electrophoresis. Sequencing was performed commercially by Biomarker Technologies (BMK) GmbH, Munster, Germany and raw paired-end reads were generated for downstream bacterial community analysis.

### 2.3. Sequence Processing and Taxonomic Annotation

Raw paired-end reads were processed by the sequencing provider using a standard 16S amplicon bioinformatics pipeline. Low-quality reads were filtered with Trimmomatic v0.33, primer/adaptor sequences were removed using Cutadapt v1.9.1 [30,31] and paired-end reads were merged, denoised and chimera filtered using DADA2 within QIIME2 [32,33]. Default/recommended parameters of the provider pipeline were used for quality filtering, paired-end merging, maximum expected error filtering, and chimera removal. Taxonomic assignment of representative ASV sequences was performed against the SILVA 138 database using a QIIME2-compatible trained classifier [34]. Before downstream bacterial community analysis, ASVs assigned to chloroplasts, mitochondria, unassigned sequences and non-bacterial lineages were removed. Extraction blanks, PCR negative controls and mock communities were not available for formal contaminant modelling; therefore, possible low level reagent or environmental contamination is acknowledged as a limitation. The final filtered ASV table was used for alpha-diversity, beta-diversity, taxonomic-composition and differential-abundance analyses and low-abundance uncertain taxa were not used as major evidence for ecological interpretation.

### 2.4. Microbial Diversity, Differential Abundance and Network Analysis

Alpha-diversity analysis was performed to evaluate within-sample bacterial richness, diversity, and phylogenetic diversity. To reduce bias caused by uneven sequencing depth, the filtered ASV table was normalized before diversity analysis using an even-depth rarefied table generated from the retained reads. Diversity metrics included observed ASVs, ACE, Chao1, Shannon diversity, Simpson diversity, Faith’s phylogenetic diversity, and Good’s coverage. Simpson diversity was reported as 1 − D, where D = Σpi^2^ and pi is the proportional abundance of each ASV. Rarefaction analysis was conducted as a sequencing-depth quality-control assessment to evaluate whether sequencing depth was sufficient to capture most of the bacterial diversity present in each sample.

Beta-diversity analysis was conducted to evaluate differences in bacterial community composition among sampling groups. Community dissimilarity was calculated using the binary Jaccard distance metric. Because binary Jaccard is based on presence/absence information, these ordination results were interpreted as reflecting ASV compositional turnover rather than abundance-weighted community differences [35]. Principal coordinate analysis (PCoA) and non-metric multidimensional scaling (NMDS) were used to visualize community-level clustering and separation patterns. Hierarchical clustering analysis was additionally performed using the unweighted pair-group method with arithmetic mean (UPGMA). The statistical significance of overall group-level differences in bacterial community composition was assessed using analysis of similarities (ANOSIM). Because of the limited number of colony-level replicates per island × habitat group, ANOSIM was interpreted as an exploratory overall test rather than as a factorial test separating island, habitat, and island × habitat effects. Statistical analyses were conducted using QIIME2, R software, and associated ecological analysis packages.

Differential-abundance screening was performed using LEfSe, STAMP and Metastats [36,37,38]. These methods were used for complementary purposes rather than as independent confirmation of the same biological effect. LEfSe was used to identify discriminant taxa and estimate linear discriminant analysis (LDA) effect sizes, STAMP was used to compare and visualize relative-abundance differences at the phylum, genus and predicted function levels and Metastats was used to evaluate genus-level abundance patterns with multiple-testing output where available. The results were summarized as candidate group associated taxa and taxa supported by only one method or showing inconsistent patterns among methods were not over-interpreted.

A supplementary exploratory microbial association network was constructed using Spearman correlation analysis among dominant bacterial genera. Statistically supported positive and negative correlations were retained to visualize possible co-occurrence patterns. Topological roles of taxa within the network were further evaluated using Zi–Pi analysis, which classified taxa as peripherals, connectors, module-hubs or network-hubs according to within module and among-module connectivity [39]. Because the network was based on 12 colony level samples and relative-abundance data, the co-occurrence and Zi–Pi analyses were interpreted strictly as preliminary visual summaries rather than robust evidence of microbial interactions or stable network organization. Also, Spearman correlations were calculated from relative-abundance data, potential compositional closure effects and spurious associations cannot be excluded; future studies with larger sample sizes should apply compositionality-aware network approaches such as SPIEC-EASI or SparCC.

### 2.5. Functional Prediction Analysis

Functional prediction of bacterial communities was performed using PICRUSt2 v2.5 based on ASV abundance profiles [40]. The proportion of ASVs that could not be assigned to predicted functional categories was calculated to indicate the annotation coverage of the functional-prediction analyses. Predicted Kyoto Encyclopedia of Genes and Genomes (KEGG) pathways and Clusters of Orthologous Groups (COG) functional categories were generated to evaluate inferred functional potential of the bacterial communities. NSTI values were not available from the provider output; therefore, PICRUSt2-based results were interpreted as exploratory functional predictions rather than validated functional profiles. Differences in predicted functional categories were evaluated using STAMP. For STAMP comparisons of predicted functional profiles, the colony-level sample was treated as the biological unit of analysis. Functional categories were compared using STAMP with multiple-testing correction where applicable.

Ecological-function prediction was additionally performed using the FAPROTAX database to infer potential microbial ecological functions associated with bacterial taxa identified in the samples. FAPROTAX was used to infer ecological functions from bacterial taxonomic profiles according to curated taxonomy–function associations [41]. Graphical visualization and statistical analyses were performed using QIIME2, R software v 4.x and associated microbiome-analysis packages. Because PICRUSt2 and FAPROTAX rely on reference genomes and curated functional annotations, and because many insect-associated bacterial taxa remain poorly represented in current databases, predicted functions were interpreted strictly as exploratory functional potential rather than measured functional activity. As FAPROTAX was primarily developed for environmental microbial communities and infers ecological functions solely from taxonomic assignments, FAPROTAX-derived ecological interpretations were treated with particular caution and were not considered direct evidence of gene content, metabolic activity or ecological function.

## 3. Results

### 3.1. Taxonomic Composition of the Gut Bacterial Microbiota of T. laeviceps

**Figure 2:**
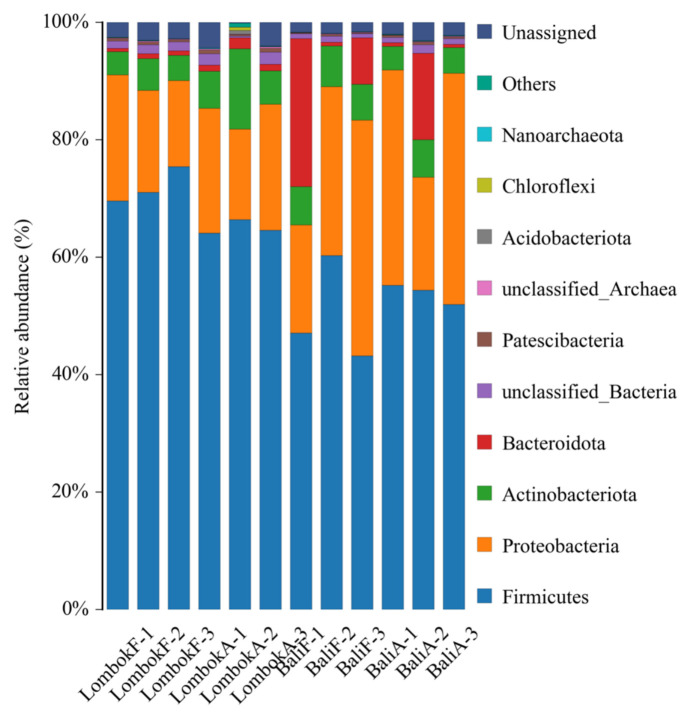
Taxonomic composition of bacterial communities in *T. laeviceps* gut samples. Stacked bar plot showing the relative abundance of dominant bacterial phyla across colonies from Bali and Lombok sampled in forest and agroforestry habitats. Less abundant taxa and unassigned sequence are grouped as “Others” and “Unassigned”, respectively. Taxonomic profiles are shown after removal of chloroplast, mitochondrial, unassigned, and non-bacterial ASVs; low-abundance uncertain categories were not used for major ecological interpretation.

The gut bacterial communities of *T. laeviceps* were dominated by several major dominant bacterial phyla after ASV filtering, with observable variation in relative abundance between Bali and Lombok colonies and among habitat groups (Figure 2). *Firmicutes* and *Proteobacteria* accounted for the largest proportions of the bacterial community across most samples, followed by *Actinobacteriota*, *Bacteroidota*, whereas other phyla occurred at comparatively low relative abundances. Lombok colonies generally showed a higher relative abundance of *Firmicutes*, particularly in forest-associated samples, whereas Bali colonies tended to show greater relative contributions of *Proteobacteria* and, in some samples, *Bacteroidota*. The same dominant bacterial phyla were broadly shared across the dataset, but their relative abundances varied among islands, habitats, and individual colonies. The taxonomic profiles also showed within-group heterogeneity, with some colonies displaying higher proportions of specific bacterial groups than others. The phylogenetic distribution of dominant taxa was consistent with the main bacterial phyla observed in the stacked abundance profiles (Figure S1).

### 3.2. Alpha Diversity and Sequencing Sufficiency

**Figure 3:**
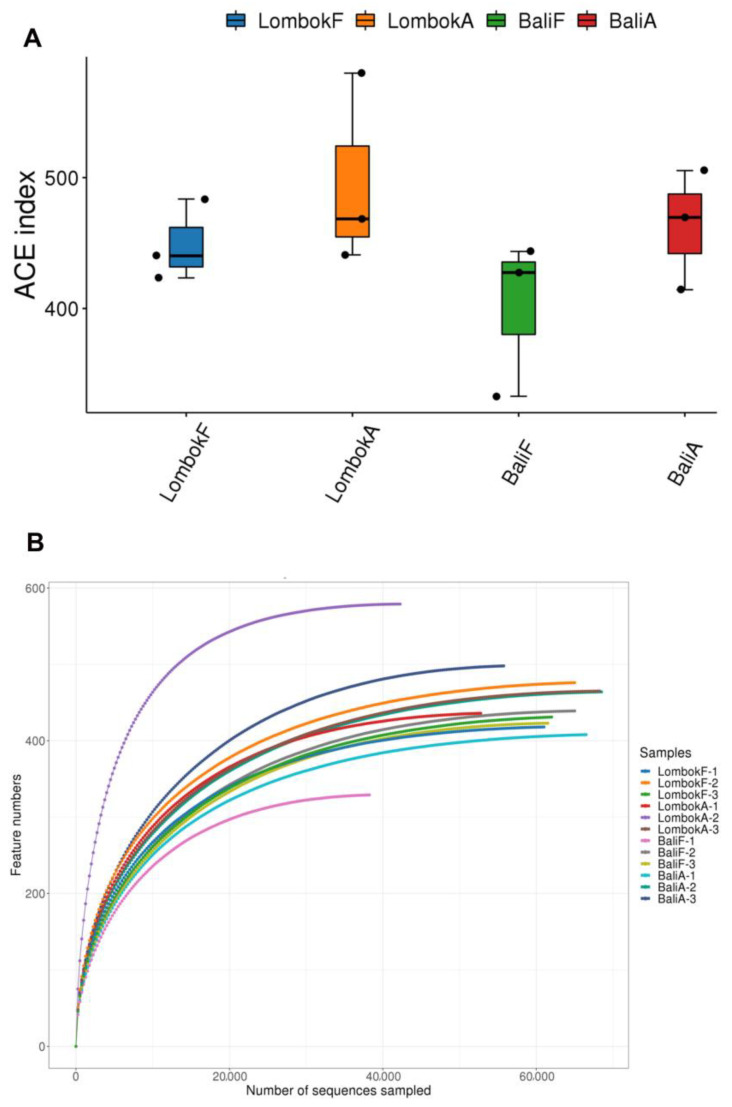
Alpha diversity and rarefaction analysis of *T. laeviceps* gut bacterial communities. (**A**) ACE richness index across the four sampling groups. (**B**) Rarefaction curves showing the number of detected ASVs as sequencing depth increased across samples.

Alpha-diversity analysis showed exploratory variation in bacterial richness and diversity among colony level samples and sampling groups (Figure 3A; Table 1). Figure 3A summarizes group level patterns, whereas Table 1 provides the individual colony level diversity values. Because each group contained only three biological replicates, alpha-diversity patterns were interpreted conservatively. Based on ACE and Chao1 richness estimators, the apparent higher richness in LombokA was strongly influenced by LombokA-2, which showed the highest observed ASV number, ACE, Chao1 and Shannon diversity values. Therefore, this pattern should be interpreted as sample influenced rather than as definitive evidence of consistently higher richness in Lombok agroforestry colonies.

BaliA and LombokF displayed intermediate richness patterns. Diversity indices also varied among samples. Shannon diversity was highest in LombokA-2, indicating pronounced sample-level heterogeneity within the LombokA group. Simpson diversity, expressed as 1 − D, showed a similar pattern, with LombokA-2 exhibiting the highest Simpson diversity value. Faith’s phylogenetic diversity also differed among samples; however, it did not fully parallel ASV richness, indicating that higher feature richness did not always correspond to broader phylogenetic breadth (Table 1).

Rarefaction analysis was used as a sequencing-depth quality-control assessment rather than as primary biological evidence (Figure 3B). Most curves gradually approached saturation, and this interpretation was further supported by the high Good’s coverage values, read-length distribution, per-sample feature counts, and Shannon rarefaction curves (Figure S2A–C). However, LombokF-1 and LombokF-2 did not show a complete plateau, suggesting that additional sequencing could potentially recover some rare ASVs in these samples. The BaliA-3 curve was also re-examined and interpreted only as part of sequencing-depth quality control.

### 3.3. Beta-Diversity Patterns Indicate Group-Associated Microbiome Structure

**Figure 4:**
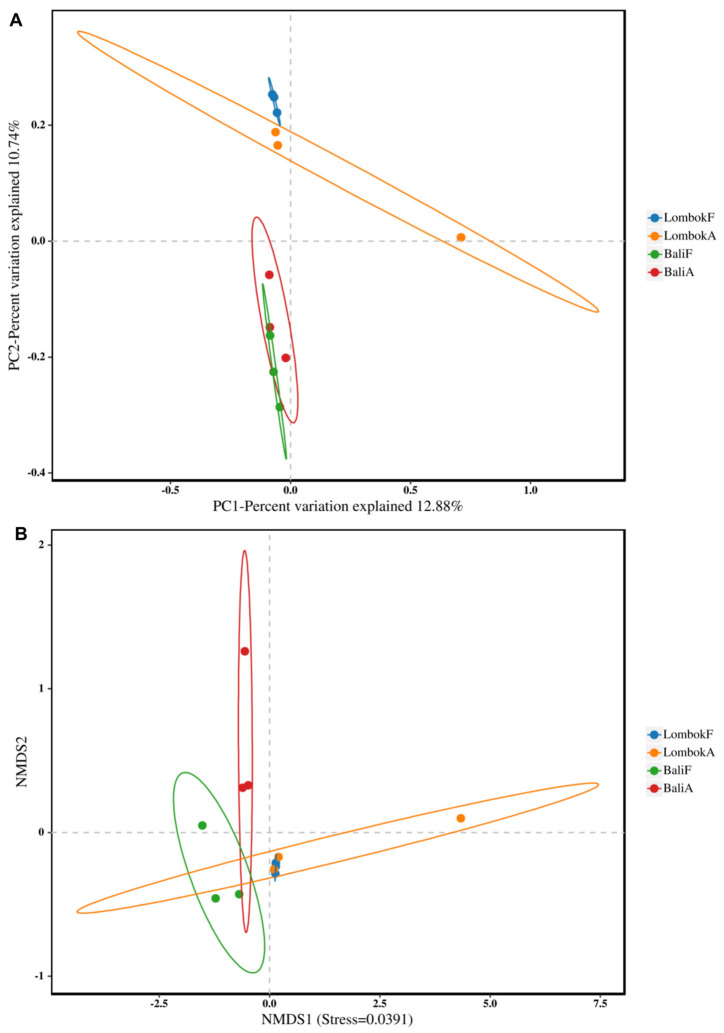
Beta-diversity ordination of *T. laeviceps* gut bacterial communities. (**A**) Principal coordinate analysis (PCoA) and (**B**) non-metric multidimensional scaling (NMDS) based on bacterial community dissimilarity among BaliF, BaliA, LombokF and LombokA samples. Ellipses indicate group-level dispersion patterns. Because each group contained only three colony level samples, ellipses are shown only as visual aids for group dispersion and should not be interpreted as statistically robust confidence regions.

Beta-diversity analyses revealed group-associated variation in bacterial community composition among the four sampling groups (Figure 4A,B). Ordination based on binary Jaccard dissimilarity showed partial separation among BaliF, BaliA, LombokF and LombokA samples, indicating that gut bacterial community structure varied across island and habitat categories. LombokF samples clustered closely together, whereas LombokA showed greater dispersion, suggesting stronger within-group heterogeneity in this group. Bali samples were positioned separately from most Lombok samples, although some overlap and sample-level variability were observed, indicating that the observed community structure reflected both group-level and colony-level variation.

**Figure 5:**
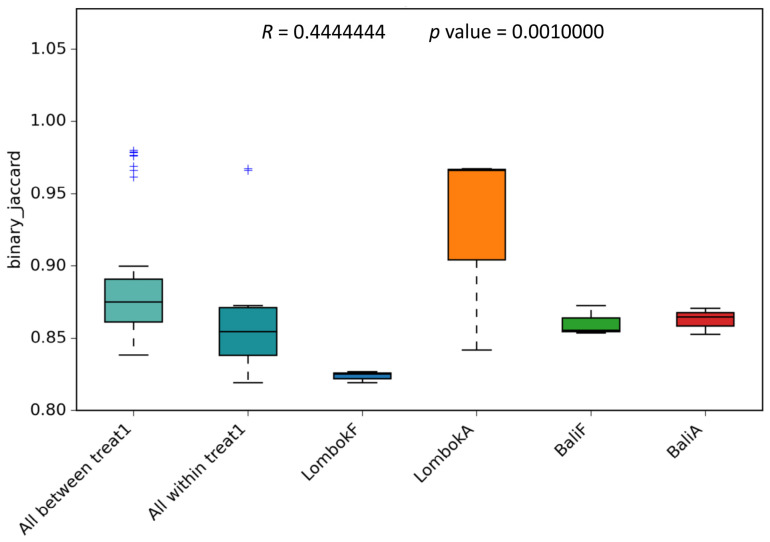
Statistical assessment of beta-diversity differences among groups. ANOSIM analysis based on binary-Jaccard distances showing within-group and between-group bacterial community dissimilarities. The analysis indicated moderate but significant group-level differentiation among bacterial communities (*R* = 0.4444, *p* = 0.001).

NMDS analysis showed a low stress value of 0.0391, indicating a good ordination fit and supporting the reliability of the visualized community relationships (Figure 4B). Statistical comparison of beta-diversity distances further supported significant group-level differences in bacterial community composition. ANOSIM based on binary Jaccard distances indicated moderate overall differentiation among the four sampling groups (*R* = 0.4444, *p* = 0.001; Figure 5). This result suggests that between-group dissimilarities were generally greater than within-group dissimilarities. However, because ANOSIM does not partition island, habitat and island × habitat interaction effects, and because group dispersion was not formally tested, this result was interpreted as evidence of overall group-associated community differentiation.

UPGMA clustering further supported non-random grouping of several samples according to island and habitat categories (Figure S3). LombokF samples clustered together, BaliF samples formed a closely related cluster, and BaliA-1 and BaliA-2 grouped together, whereas LombokA-2 and BaliA-3 appeared more distinct from their respective group clusters. Together, these analyses indicate that the gut bacterial microbiota of *T. laeviceps* showed significant, though incomplete, group-associated differentiation across the Bali and Lombok populations and between habitat categories.

### 3.4. Candidate Bacterial Taxa Associated with Island and Habitat Groups

**Figure 6:**
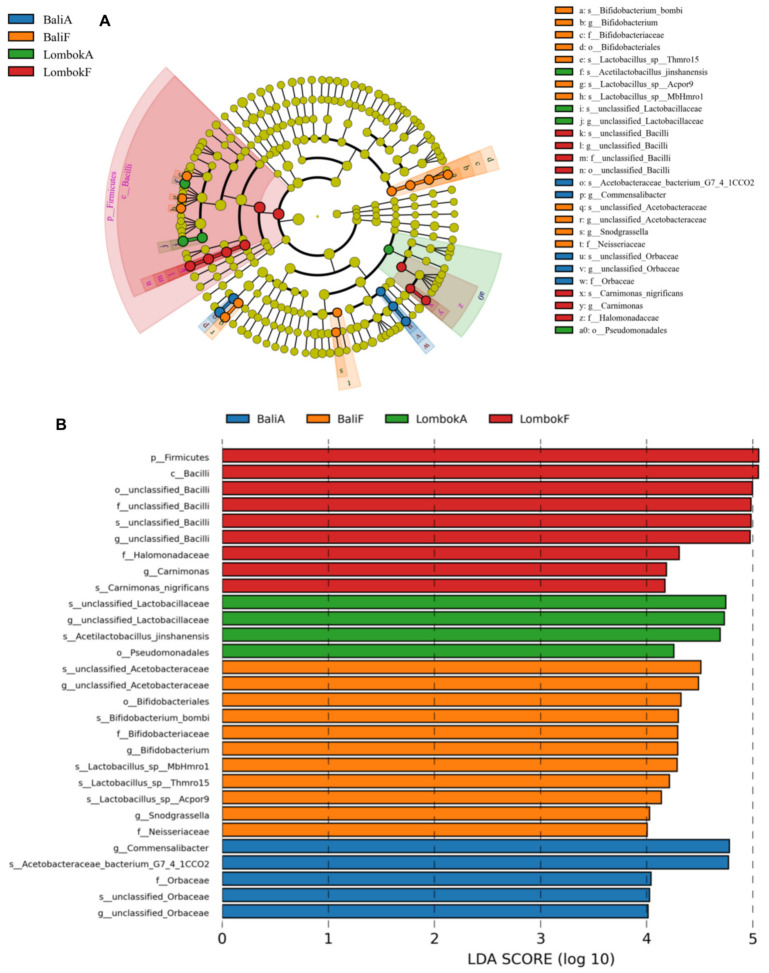
Differential bacterial taxa identified by LEfSe analysis. (**A**) LEfSe cladogram showing bacterial taxa associated with each sampling group. Colored nodes indicate putatively enriched taxa in specific groups, whereas non-discriminant taxa are shown in yellow. (**B**) Linear discriminant analysis showing discriminant taxa ranked by LDA score.

LEfSe analysis identified several candidate discriminatory taxa that differed among sampling groups based on relative abundance and LDA effect size (Figure 6). In the LEfSe analysis, discriminant taxa were distributed across several taxonomic ranks, suggesting that group-associated microbiome variation involved coordinated shifts across multiple bacterial clades (Figure 6A). Because of the limited sample size, LEfSe results were interpreted as exploratory candidate discriminant patterns rather than confirmed biomarkers. The LDA score distribution further ranked these taxa according to their contribution to group differentiation (Figure 6B). Taxa with higher LDA scores were interpreted as candidate discriminant taxa associated with specific island–habitat groups, rather than as causal drivers of community divergence. LEfSe identified group-associated enrichment patterns across the four sampling groups. LombokF-associated taxa included Firmicutes, Bacilli and several unclassified Bacilli-related lineages, whereas LombokA-associated taxa included unclassified *Lactobacillaceae*, *Acetilactobacillus jinshanensis* and *Pseudomonadales*. BaliF-associated taxa included *Bifidobacterium*-related taxa, Lactobacillus-associated taxa, unclassified *Acetobacteraceae*, *Snodgrassella* and *Neisseriaceae*, while BaliA associated taxa included *Commensalibacter*, an *Acetobacteraceae*-related bacterium and unclassified *Orbaceae*. These taxa were interpreted as putatively enriched bacterial groups rather than validated biomarkers. The observed differences suggest possible group-associated taxonomic patterns, but they do not demonstrate coordinated ecological shifts across bacterial clades or causal relationships among taxa.

**Figure 7:**
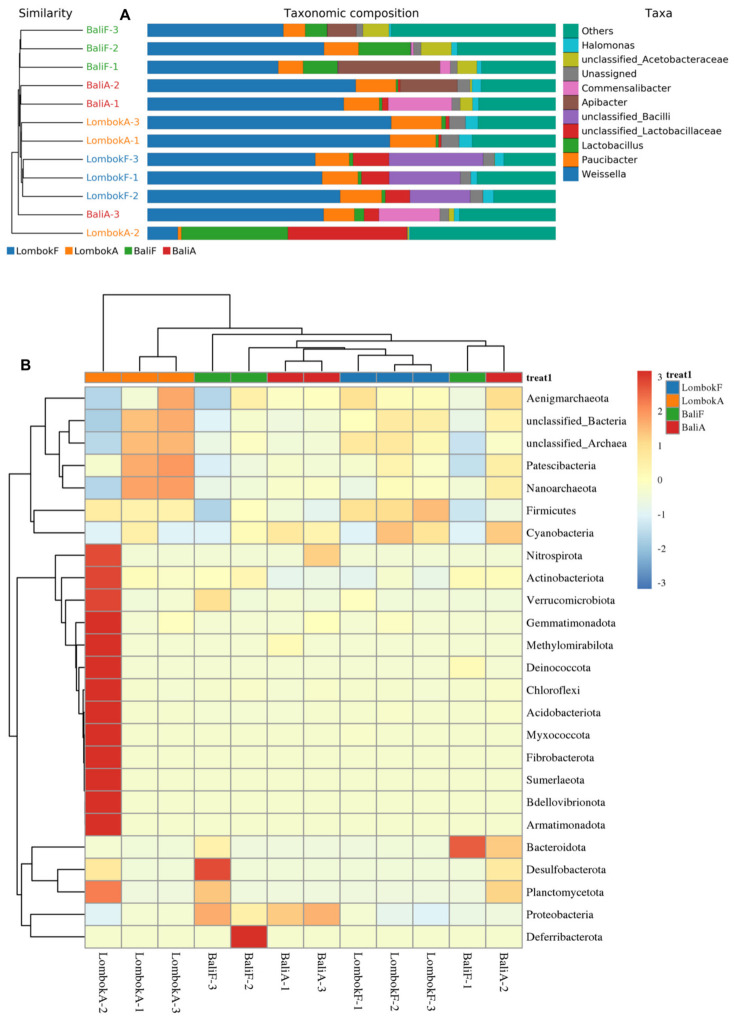
Taxonomic clustering and abundance patterns across samples. (**A**) Combined UPGMA clustering tree and genus-level taxonomic composition across samples. (**B**) Heatmap of phylum-level relative abundance showing sample clustering and group-associated taxonomic variation.

The combined clustering and taxonomic abundance analysis further illustrated how genus-level community composition contributed to sample relationships (Figure 7A). Samples with similar genus-level abundance profiles tended to occur on neighboring branches of the UPGMA tree, indicating that clustering patterns were partly driven by shared dominant bacterial genera. However, some samples did not cluster tightly with their respective groups, suggesting that colony-level variation also contributed to the observed community structure.

The taxonomic heatmap provided an additional view of group-associated variation in relative abundance at the phylum level (Figure 7B). LombokF samples showed relatively consistent clustering, whereas BaliA and LombokA samples showed greater dispersion, indicating higher within-group heterogeneity. These clustering patterns were broadly consistent with the ordination analyses, showing that community-level separation was reflected in taxon-level abundance variation.

**Figure 8:**
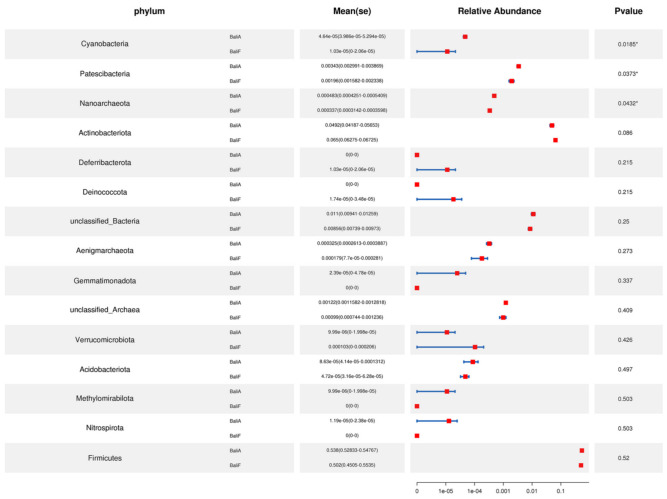
Phylum–level differences between Bali forest and Bali agroforestry samples. STAMP comparison showing relative abundance differences and confidence intervals for bacterial phyla between BaliF and BaliA groups. This pairwise comparison is presented as an exploratory within-island habitat contrast and should not be interpreted as a full factorial test of habitat effects. Low-abundance categories with uncertain biological interpretation should be considered exploratory and were not used as primary evidence for habitat-associated bacterial differences. Asterisks (*) indicate significant differences between samples.

STAMP analysis showed that BaliF and BaliA differed in the relative abundance of several bacterial phyla (Figure 8). The comparison plot displayed the proportional abundance of each phylum, the estimated between-group difference, and confidence intervals. *Cyanobacteria*, *Patescibacteria* and *Nanoarchaeota* showed nominal differences between BaliF and BaliA, whereas other phyla showed weaker or non-significant differences. These results suggest that the BaliF–BaliA comparison suggested possible habitat-associated taxonomic variation within Bali at broad taxonomic levels, although the magnitude and direction of differences varied among phyla. Because the study included four island × habitat groups, pairwise STAMP results were not used as the primary basis for overall inference. The BaliF versus BaliA comparison was retained as an illustrative within-island habitat contrast, because both groups originated from Bali and differed mainly in habitat category.

**Figure 9:**
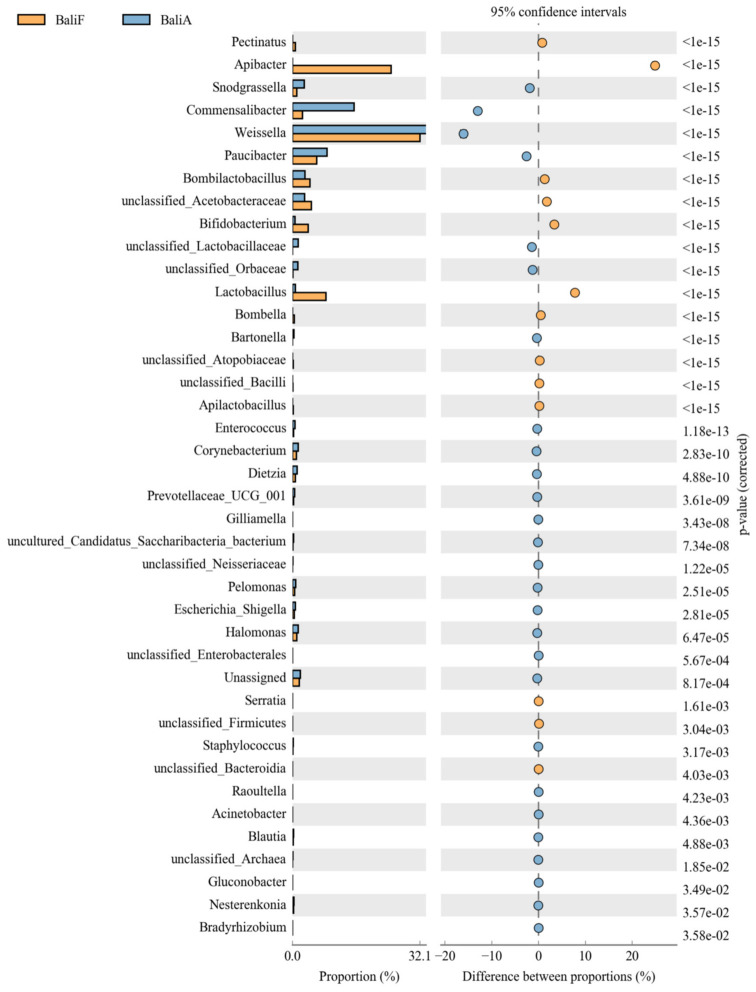
Genus-level differential abundance between Bali forest and Bali agroforestry samples. STAMP analysis showing genus-level proportional abundance, between-group differences, 95% confidence intervals and corrected significance values for dominant bacterial genera. This analysis was used to illustrate candidate genus-level differences between two Bali habitat categories.

At the genus-level, STAMP analysis provided a more detailed view of bacterial taxa contributing to differences between BaliF and BaliA samples (Figure 9). Compared with the phylum-level analysis, the genus-level results resolved specific bacterial groups whose relative abundance differed between the two Bali habitats. Our results shows the proportional abundance in each group, the between-group difference, 95% confidence intervals, and corrected *p*-values, allowing direct comparison of the magnitude and reliability of each difference. Several genera showed differential proportional abundance between the two Bali habitat categories, indicating that habitat-associated variation involved specific bacterial groups rather than only broad phylum-level shifts. These genus-level patterns help explain the partial separation of BaliF and BaliA samples observed in the ordination and clustering analyses, despite both groups originating from the same island.

Metastats analysis identified selected bacterial genera showing nominal abundance signals across the four sampling groups (Table 2). These genera included *Bifidobacterium*, *Bombella*, unclassified_*Acetobacteraceae*, unclassified_*Bacilli*, *Lactobacillus*, *Pseudonocardia*, *Apilactobacillus*, *Nodosilinea*_PCC_7104 and *Enterococcus*. Among these taxa, *Bifidobacterium* showed the highest relative abundance in LombokF, followed by LombokA and BaliF, whereas BaliA showed the lowest abundance. *Lactobacillus*, *Bombella*, unclassified_*Acetobacteraceae*, unclassified_*Bacilli* and *Apilactobacillus* were more abundant in BaliF than in BaliA, while *Enterococcus* and *Nodosilinea*_PCC_7104 were relatively more abundant in BaliA. *Pseudonocardia* showed its highest relative abundance in LombokA. However, because the corresponding *q*-values were high after multiple testing correction, these patterns were interpreted as exploratory candidate signals rather than statistically confirmed differential abundance. To summarize group associated taxonomic variation, LEfSe, STAMP and Metastats were used as complementary differential abundance screening approaches. LEfSe highlighted discriminatory taxa with LDA effect sizes, STAMP visualized relative-abundance differences and Metastats provided genus-level statistical support where available. Therefore, the results are presented as an integrated summary of candidate group-associated taxa rather than as separate confirmatory analyses.

### 3.5. Predicted Functional Profiles and Microbial Association Patterns

**Figure 10:**
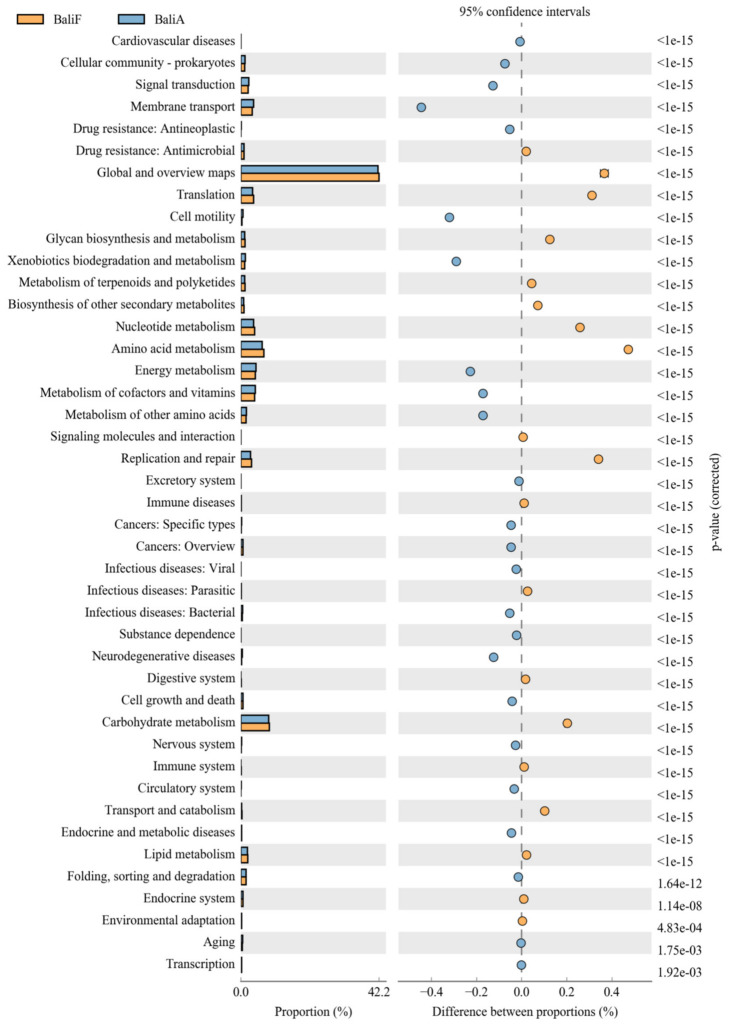
Predicted KEGG functional profiles of *T. laeviceps* gut bacterial communities. STAMP comparison of PICRUSt2-predicted KEGG functional categories showing proportional abundance, between-sample or between-group differences, 95% confidence intervals, and corrected significance values for predicted pathway categories. Because these functions were predicted from 16S rRNA profiles and each group contained limited biological replication, the statistical values are shown as exploratory indicators rather than definitive evidence of functional differences.

Exploratory PICRUSt2-based functional prediction revealed that the taxonomic variation observed in *T. laeviceps* gut bacterial communities was accompanied by variation in inferred functional potential (Figure 10). PICRUSt2-based KEGG functional prediction showed differences in the proportional abundance of several predicted functional categories between the compared samples. STAMP comparison suggested exploratory differences in the relative abundance of predicted functional categories. These patterns suggest that differences in bacterial community composition were reflected in predicted functional profiles, although the overall functional structure remained broadly conserved. The broader KEGG pathway overview showed that many predicted functions were shared across the dataset, but their relative abundance patterns differed among colonies and bacterial taxa (Figure S4). Functional categories related to metabolism, environmental information processing, cellular processes and genetic information processing were consistently represented. This indicates that the gut bacterial communities retained a shared predicted functional core, while still exhibiting variation in the proportional representation of individual functional categories.

The STAMP comparison identified differences in functions associated with chemo-heterotrophy, aerobic chemo-heterotrophy, fermentation, nitrate reduction, nitrite respiration, nitrite ammonification, and nitrogen respiration. Functions such as fermentation and chemo-heterotrophy represented major predicted ecological activities, whereas several nitrogen-related functions occurred at lower relative abundance. These results suggest that taxonomic differences among bacterial communities were accompanied by changes in predicted ecological-function profiles. The COG functional overview also showed that predicted gene categories related to metabolism, cellular processing, information storage and signaling functions were broadly distributed across the dataset, although their proportional representation varied among groups (Figure S5). Similar to the KEGG analysis, the COG profiles indicated that the microbiomes shared many conserved functional categories while exhibiting group-associated variation in predicted functional composition.

A supplementary exploratory co-occurrence network was generated to visualize possible associations among dominant bacterial genera (Figure S6). Because this analysis was based on only 12 colony-level samples and Spearman correlations of relative abundance data, the inferred associations should be interpreted strictly as preliminary visual patterns rather than evidence of direct microbial interactions. The Zi–Pi analysis was similarly used only as an exploratory description of possible topological roles (Figure S7). Because Zi–Pi classification depends on the stability of the underlying correlation network, these topological roles were interpreted only as exploratory descriptors and not as confirmed ecological roles of bacterial taxa. Predicted ecological function analysis using FAPROTAX further revealed variation in inferred ecological functions (Figure S8). FAPROTAX predicted several putative ecological function categories among the sampled colonies; however, because FAPROTAX infers functions solely from taxonomic assignments and was primarily developed for environmental microbial communities, these results were presented mainly as Supplementary Materials.

## 4. Discussion

Microbiomes associated with social bees are increasingly recognized as integral components of pollinator biology, influencing nutrition, metabolism, immunity, pathogen resistance, and ecological adaptation [42,43,44]. However, despite rapid advances in insect microbiome research, comparatively little is known about how major biogeographic transitions influence microbial community assembly in tropical pollinators [45]. The present study shows that the gut bacterial microbiota of *T. laeviceps* differed between colonies sampled from Bali and Lombok, with additional variation associated with forest and agroforestry habitats. Because the study was based on limited colony-level replication and several analyses relied on 16S rRNA-based inference, the results are interpreted as descriptive evidence of island and habitat associated microbiome variation rather than as definitive evidence of causal ecological mechanisms.

The bacterial communities detected in *T. laeviceps* were dominated by Firmicutes, Proteobacteria, Actinobacteriota and Bacteroidota, indicating that the gut microbiota of this stingless bee is largely composed of bacterial groups commonly reported from social bees and other insect-associated microbiomes. Moreover, several core gut bacterial taxa have been consistently identified across Apis, Bombus and Meliponini bees, supporting the idea that social bees maintain relatively conserved microbial assemblages despite geographic separation and ecological variability [17]. Several bee-associated bacterial lineages, including *Lactobacillus*, *Apilactobacillus*, *Bifidobacterium*, *Bombella*, *Commensalibacter* and Acetobacteraceae-related taxa, have been frequently associated with sugar-rich bee niches, carbohydrate metabolism, fermentation, pollen-derived substrate processing, and gut homeostasis [27,46,47,48]. The presence of these taxa across Bali and Lombok suggests that *T. laeviceps* maintains a broadly conserved social-bee-associated bacterial framework. However, the relative abundance of these bacterial groups varied among islands, habitats and colonies, indicating that microbiome divergence was driven primarily by the restructuring of shared community members rather than the complete replacement of dominant bacterial lineages. These taxonomic patterns suggest that both island/habitat context and colony-level variation may contribute to gut bacterial community structure, although these factors could not be fully separated because of the present sampling design.

This pattern is ecologically important because host-associated microbiomes often diverge through changes in the proportional abundance of shared taxa, rather than through total community turnover [45]. Similar patterns have been reported in honeybees and bumble bees, where core or recurrent bacterial taxa may persist across populations but shift in abundance in response to local diet, environmental exposure, host condition and ecological context [49,50]. In the present study, beta-diversity analyses indicated significant group-associated differentiation among *T. laeviceps* gut bacterial communities, with Bali and Lombok samples showing separation despite the relatively short geographic distance between the islands. Because the deep Lombok Strait has historically limited terrestrial connectivity between Bali and Lombok, the Wallace Line provides a compelling biogeographic framework for interpreting these patterns [3,51]. However, because binary Jaccard distance is based on presence/absence information, these patterns should be interpreted as ASV compositional turnover rather than abundance-weighted community differences. Nevertheless, the observed microbiome divergence should be interpreted as compatible with island-associated structuring within a Wallace Line biogeographic context, rather than as direct evidence that the Wallace Line alone caused the microbial differences.

Several non-mutually exclusive hypotheses may explain the observed microbiome divergence: First, regional environmental microbial pools may differ between Bali and Lombok as a result of long-term ecological separation, differences in vegetation and local environmental conditions [51]. Stingless bees interact continuously with environmental microbial sources through floral visitation, nectar and pollen collection, resin gathering, nest materials, soil contact and social exchange within colonies. Therefore, differences in floral resources, nesting substrates, and environmental bacterial reservoirs may contribute to the assembly of gut bacterial communities. Second, host population structure may influence microbial filtering through host genotype, immune regulation, gut physicochemical conditions and socially transmitted bacterial lineages. Third, island-associated differences in land use, vegetation composition, and ecological disturbance may further shape microbial exposure and resource availability [17,45,50]. However, these variables were not directly measured in the present study; therefore, they should be interpreted as possible explanatory factors rather than confirmed mechanisms. An additional possibility is that host population structure contributed to the observed microbiome differences. Tetragonula in Southeast Asia is taxonomically complex and geographically separated colonies may differ genetically or morphologically. Because host genetic variation was not assessed in this study, we cannot exclude the possibility that differences in gut microbiota partly reflect host population structure rather than island environment alone. Future studies combining microbiome profiling with host genetic or morphometric analyses will be necessary to separate environmental effects from host-associated filtering.

Within-island habitat variation also appeared to contribute to microbiome differentiation, particularly between forest and agroforestry colonies. Habitat type can influence pollinator microbiomes by altering floral diversity, nutritional resources, pesticide exposure, nesting substrates, and environmental microbial reservoirs [50,52]. Agroforestry systems generally retain greater ecological complexity than intensive monoculture systems and may preserve diverse plant resources that support pollinator foraging and microbial acquisition pathways [53]. In this study, possible habitat-associated differences were observed at both phylum and genus levels, especially within Bali. However, these habitat effects appeared secondary to the broader island-associated patterns, suggesting that both historical biogeographic context and con-temporary habitat conditions may jointly structure the gut microbiota of *T. laeviceps*. This interpretation is consistent with the view that pollinator microbiomes are shaped by multiple ecological filters operating across spatial scales, from local foraging environments to broader regional biogeography. Furthermore, alpha-diversity patterns varied among samples and groups; however, the apparent higher richness in the Lombok agroforestry group was strongly influenced by one colony level sample [54,55,56]. Therefore, these results are presented as descriptive colony and group level variation, rather than as evidence that agroforestry habitats consistently support higher gut bacterial richness.

Differential-abundance analyses identified candidate group associated taxa associated with particular island or habitat groups, including *Bifidobacterium*, *Bombella*, *Lactobacillus*, *Apilactobacillus*, *Neokomagataea*, *Pseudonocardia* and Acetobacteraceae-related lineages, but these patterns were considered as taxonomic screening results rather than definitive evidence of fixed microbial biomarkers [57,58,59]. Acetobacteraceae-related bacteria are particularly common in sugar-rich environments and have been repeatedly detected in nectar, honey and bee associated niches. Variation in their abundance may therefore reflect differences in floral resource composition, dietary substrates, or environmental acquisition routes between islands and habitats [47]. Together, the differential taxa identified here provide exploratory evidence that island and habitat-associated taxonomic variation may involve both common bee-associated bacteria and lower abundance bacterial lineages.

Predicted functional profiles suggested possible variation in inferred microbial functional potential among groups. In the main text, PICRUSt2 indicated putative differences in predicted pathways related to microbial metabolism and associated cellular processes. However, these predictions were inferred from 16S rRNA profiles and reference based databases rather than directly measured by shotgun metagenomics, meta-transcriptomics, metabolomics or culture based assays. Therefore, these results were used only to describe predicted functional potential, not direct functional activity or metabolic differences [40,41,57]. Additional taxonomy-based ecological-function predictions from FAPROTAX are provided in the Supplementary Materials and were not used as a basis for the main biological interpretation.

The microbial association network provided a preliminary visualization of possible associations among dominant bacterial genera. However, because the network was based on Spearman correlations of relative abundance data from only 12 colony-level samples, the inferred associations may be affected by compositional closure effects, spurious correlations and limited network stability. Therefore, the co-occurrence network should not be interpreted as evidence of direct microbial interactions. Similarly, Zi–Pi topological analysis depends on the stability of the underlying correlation network; therefore, taxa classified as peripherals, connectors, module hubs, or network hubs were interpreted only as exploratory descriptors of possible network topology, not as confirmed ecological roles of bacterial taxa. Future studies with larger sample sizes should apply compositionality-aware network approaches, such as SPIEC-EASI or SparCC, to test whether these preliminary association patterns are reproducible [39,60,61,62].

Although the present study focused on a targeted comparison of colonies from two islands and two habitat types, broader geographic sampling across Wallacea, together with metagenomics, metabolomics, host-genetic and environmental microbiome analyses, would provide deeper insight into the ecological and evolutionary mechanisms underlying microbiome divergence in tropical pollinators. Because each side of the Wallace Line was represented by only one island, island identity and side of the Wallace Line were not independent factors in the present design. Second, all colonies were sampled in November 2025; therefore, the study represents a single seasonal snapshot. Seasonal changes in flowering phenology, nectar and pollen availability and foraging resources may influence gut microbiota composition in bees. Third, local environmental variables such as detailed floral composition, pesticide exposure, nesting substrates, elevation, and environmental microbial reservoirs were not fully measured. Therefore, the observed island and habitat-associated patterns should be interpreted within this temporal context. Future studies including repeated seasonal sampling are needed to test the stability of these microbiome patterns over time. Overall, this study provides evidence that the gut bacterial microbiota of *T. laeviceps* varies between Bali and Lombok and is additionally shaped by habitat context. The findings extend current understanding of stingless bee microbiomes by placing them within an island-biogeographic framework. More broadly, they suggest that host-associated microbial communities may represent an underexplored microbial component of island-associated biodiversity patterns, complementing the well-established macro-ecological patterns associated with the Wallace Line.

## 5. Conclusions

In conclusion, our study detected differences in the gut microbiota of *T. laeviceps* colonies sampled from Bali and Lombok, two islands situated on opposite sides of the Wallace Line. Although Bali and Lombok colonies shared many dominant bacterial groups typical of social bees, differences in bacterial community composition, candidate differential taxa, exploratory predicted functional profiles, and microbial association patterns indicate that gut microbiome structure varied across island and habitat contexts. The gut microbiota of *T. laeviceps* differed among colonies sampled from Bali and Lombok, two islands situated on opposite sides of the Wallace Line; however, these differences should be interpreted as compatible with island-associated structuring, not as direct evidence of an independent Wallace Line effect. These findings expand current understanding of stingless bee microbiomes and suggest that host-associated microbial communities may represent an underexplored microbial dimension of tropical island biogeography. More broadly, this study highlights stingless bees as valuable systems for investigating how historical biogeographic boundaries and contemporary habitat conditions may jointly shape pollinator-associated microbiomes.

## Figures and Tables

**Table 1:** Alpha-diversity indices of *T. laeviceps* gut bacterial communities.

Sample Name	Feature	ACE	Chao1	Simpson	Shannon	PD_Whole_Tree	Coverage
BaliA-1	408	414.3381	409.7436	0.7694	3.6778	59.5214	0.9997
BaliA-2	464	469.5528	465.2121	0.7494	3.6758	63.7532	0.9998
BaliA-3	498	505.3358	499.275	0.8119	4.0872	54.1488	0.9997
BaliF-1	329	332.9081	329.8354	0.8454	4.0177	45.5812	0.9997
BaliF-2	439	443.5891	439.8041	0.8198	4.1549	59.7127	0.9998
BaliF-3	423	427.4474	423.6286	0.8861	4.5787	54.7595	0.9998
LombokA-1	436	440.9565	437.0825	0.6835	3.5416	65.4348	0.9997
LombokA-2	579	579.7958	579.0423	0.9315	5.6149	65.9008	0.9999
LombokA-3	465	468.4518	465.4091	0.6787	3.4944	66.8049	0.9999
LombokF-1	418	423.3846	419.3636	0.8174	4.0897	61.5345	0.9998
LombokF-2	476	481.4325	477.8325	0.8724	3.3924	60.6875	0.9976
LombokF-3	431	437.4538	433.5432	0.7147	3.8857	69.5478	0.9990

Alpha diversity indices were calculated using QIIME2. Features values represent the number of detected ASVs. ACE and Chao1 estimate bacterial richness, Shannon and Simpson indices represent diversity/evenness, PD_whole_tree represents Faith’s phylogenetic diversity, and coverage represents Good’s coverage. Values are presented for individual colony level pooled samples; group-level summaries are shown in Figure 3A. Simpson represents Simpson’s diversity index expressed as 1 − D, where D = Σpi^2^ and pi is the proportional abundance of each ASV.

**Table 2:** Selected bacterial genera showing nominal abundance differences across the four sampling groups based on Metastats analysis.

Genus	Bali A	Bali F	Lombok A	Lombok F	*p*	*q*
*Bifidobacterium*	1.12 × 10^−2^	3.95 × 10^−2^	5.21 × 10^−2^	9.40 × 10^−2^	2.12 × 10^−3^	0.45
*Bombella*	3.28 × 10^−4^	4.00 × 10^−3^	1.10 × 10^−3^	6.20 × 10^−4^	7.76 × 10^−3^	0.78
unclassified_Acetobacteraceae	1.58 × 10^−2^	6.21 × 10^−2^	3.10 × 10^−2^	2.05 × 10^−2^	8.44 × 10^−3^	0.78
unclassified_Bacilli	5.31 × 10^−5^	1.34 × 10^−3^	8.00 × 10^−4^	1.10 × 10^−4^	9.12 × 10^−3^	0.78
*Lactobacillus*	1.21 × 10^−2^	8.84 × 10^−2^	4.50 × 10^−2^	3.10 × 10^−2^	1.32 × 10^−2^	0.81
*Pseudonocardia*	7.48 × 10^−4^	4.85 × 10^−4^	1.90 × 10^−3^	8.50 × 10^−4^	1.59 × 10^−2^	0.81
*Apilactobacillus*	7.29 × 10^−5^	1.27 × 10^−3^	9.10 × 10^−4^	1.20 × 10^−3^	1.95 × 10^−2^	0.81
*Nodosilinea_*PCC-7104	4.64 × 10^−5^	0	1.20 × 10^−5^	0	1.44 × 10^−3^	0.45
*Enterococcus*	6.91 × 10^−3^	3.66 × 10^−3^	5.10 × 10^−3^	4.20 × 10^−3^	1.88 × 10^−2^	0.81

Values represent the mean relative abundance of each genus in the four sampling groups. The *p*-value indicates statistical significance in the Metastats analysis and the *q*-value represents the corresponding multiple-testing-corrected value. Because *q*-values were high after multiple testing correction, these genera should be interpreted as nominal *p*-value-based signals and exploratory candidate taxa, not as statistically confirmed differentially abundant genera.

## Data Availability

The original contributions presented in this study are included in the article/Supplementary Materials. The raw 16S rRNA amplicon sequencing reads generated in this study have been deposited in the NCBI Sequence Read Archive under BioProject accession number PRJNA1491228. Further inquiries can be directed to the corresponding authors.

## Supplementary Materials

The following supporting information can be downloaded at: https://www.mdpi.com/article/10.3390/insects17070733/s1, Figure S1. Phylogenetic distribution of dominant bacterial taxa in the gut microbiota of *T. laeviceps*. Circular phylogenetic tree showing representative abundant bacterial ASVs detected across Bali and Lombok Forest and agroforestry samples. Inner labels indicate bacterial taxa or ASV-level representatives, colored clades indicate phylum-level classification, and outer rings indicate the distribution of taxa among sampling groups. Figure S2. Sequencing depth and diversity saturation. (A) Length distribution of quality-filtered reads. (B) Number of detected ASVs per sample. (C) Shannon rarefaction curves showing diversity saturation across sequencing depth. Figure S3. UPGMA clustering of bacterial communities. Hierarchical clustering tree shows similarity relationships among *T. laeviceps* gut bacterial communities based on bacterial community composition. Figure S4. Overview of predicted KEGG pathway composition. Stacked bar plot showing PICRUSt2-predicted KEGG functional categories across bacterial taxa. Figure S5. Overview of predicted COG functional composition. Stacked bar plot showing predicted COG functional categories across bacterial taxa based on 16S-derived functional inference. Figure S6. Co-occurrence network of bacterial genera. Spearman correlation-based network showing associations among dominant bacterial genera. Node size represents relative abundance, node color indicates phylum and edge color indicates correlation direction. Figure S7. Zi–Pi classification of network nodes. Within-module connectivity and among-module connectivity plot showing the topological roles of bacterial genera in the co-occurrence network. Figure S8. Predicted ecological functions of bacterial communities. FAPROTAX based comparison of predicted ecological functions showing proportional abundance, between sample or between group differences, 95% confidence intervals and corrected significance values for major inferred ecological-functional categories. FAPROTAX results are taxonomy based ecological function predictions and should be interpreted as exploratory, not as direct measurements of microbial activity or metabolism.

## Abbreviations

The following abbreviations are used in this manuscript:

ACE	Abundance-based Coverage Estimator
ANOSIM	Analysis of Similarities
ASV	Amplicon Sequence Variant
COG	Clusters of Orthologous Groups
DADA2	Divisive Amplicon Denoising Algorithm 2
FAPROTAX	Functional Annotation of Prokaryotic Taxa
FDR	False Discovery Rate
KEGG	Kyoto Encyclopedia of Genes and Genomes
LDA	Linear Discriminant Analysis
LEFSe	Linear Discriminant Analysis Effect Size
NMDS	Non-metric Multidimensional Scaling
SRA	Sequence Read Archive
STAMP	Statistical Analysis of Metagenomic Profiles
UPGMA	Unweighted Pair Group Method with Arithmetic Mean
QIIME2	Quantitative Insights into Microbial Ecology 2
PCoA	Principal Coordinates Analysis
