# Supplementary material for: Biogeographic and Habitat-Associated Variation in the Gut Microbiota of the Stingless Bee Tetragonula laeviceps Between Bali and Lombok, Two Islands Situated on Opposite Sides of the Wallace Line
Source: Insects. 2026 Jul 16;17(7):733. doi: 10.3390/insects17070733 (PMC13411446; doi:10.3390/insects17070733)
Supplement: Supplementary file 1 [file insects-17-00733-s001.zip › insects-4397864-supplementary.pdf]

Supporting information

Biogeographic and habitat-associated variation in the gut mi-crobiota of the stingless bee Tetragonula laeviceps between Bali and Lombok, two islands situated on opposite sides of the Wallace Line

The following supporting information is available for this article:

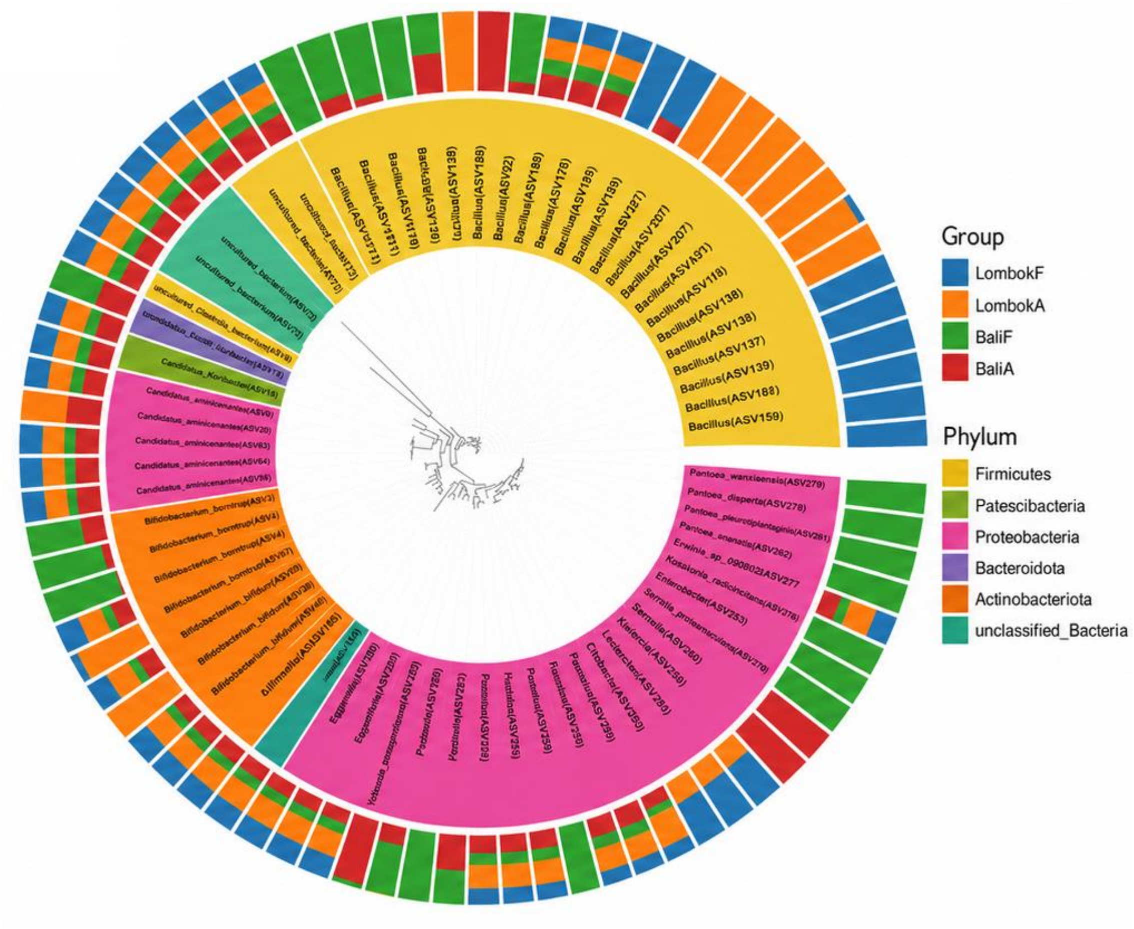

Figure S1. Phylogenetic distribution of dominant bacterial taxa in the gut microbiota of *T. laeviceps*. Circular phylogenetic tree showing representative abundant bacterial ASVs detected across Bali and Lombok Forest and agroforestry samples. Inner labels indicate bacterial taxa or ASV-level representatives, colored clades indicate phylum-level classification, and outer rings indicate the distribution of taxa among sampling groups.

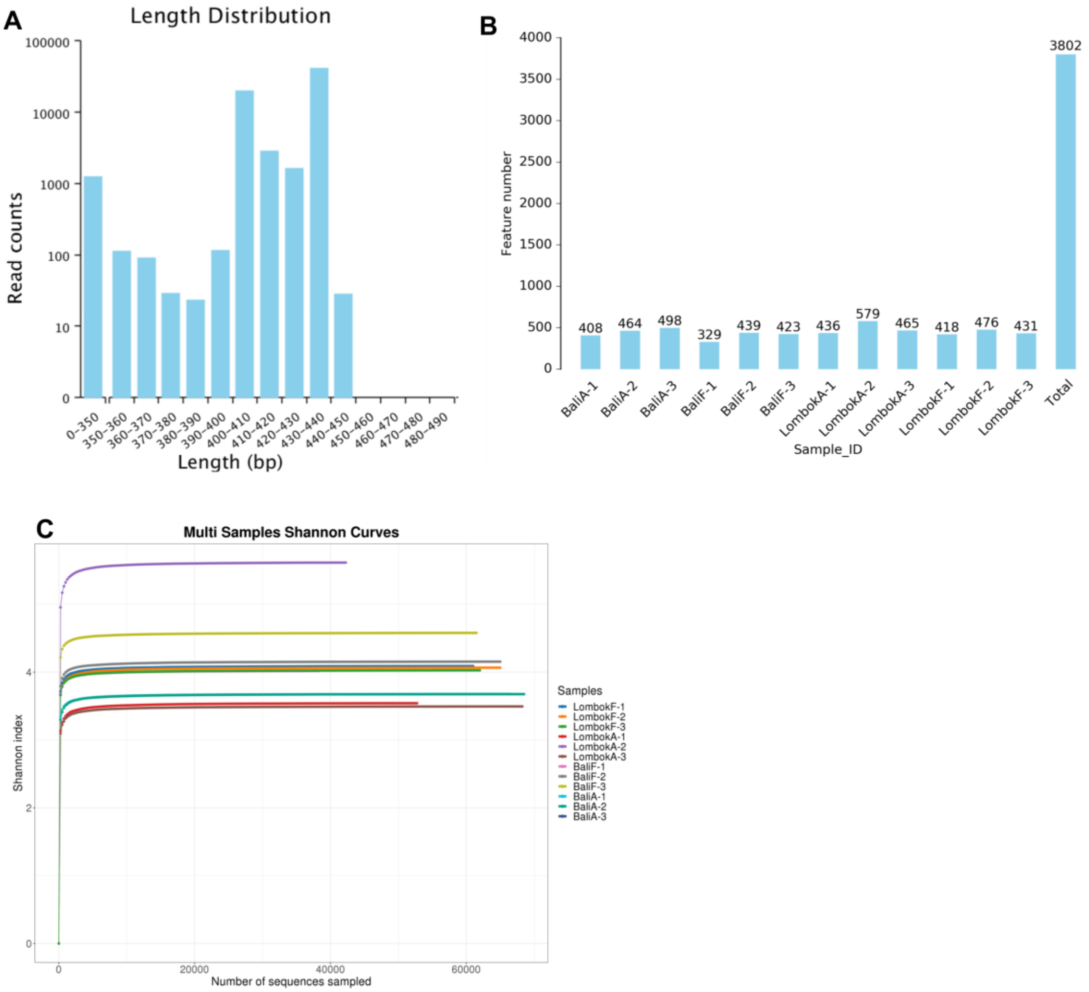

Figure S2. Sequencing depth and diversity saturation. (**A**) Length distribution of quality-filtered reads. (**B**) Number of detected ASVs per sample. (**C**) Shannon rarefaction curves showing diversity saturation across sequencing depth.

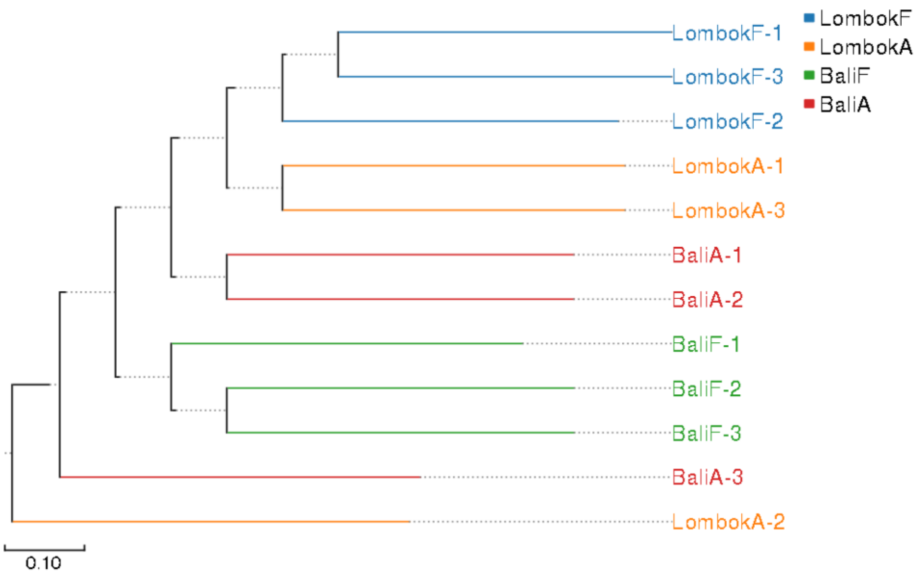

Figure S3. UPGMA clustering of bacterial communities. Hierarchical clustering tree shows similarity relationships among *T. laeviceps* gut bacterial communities based on bacterial community composition.

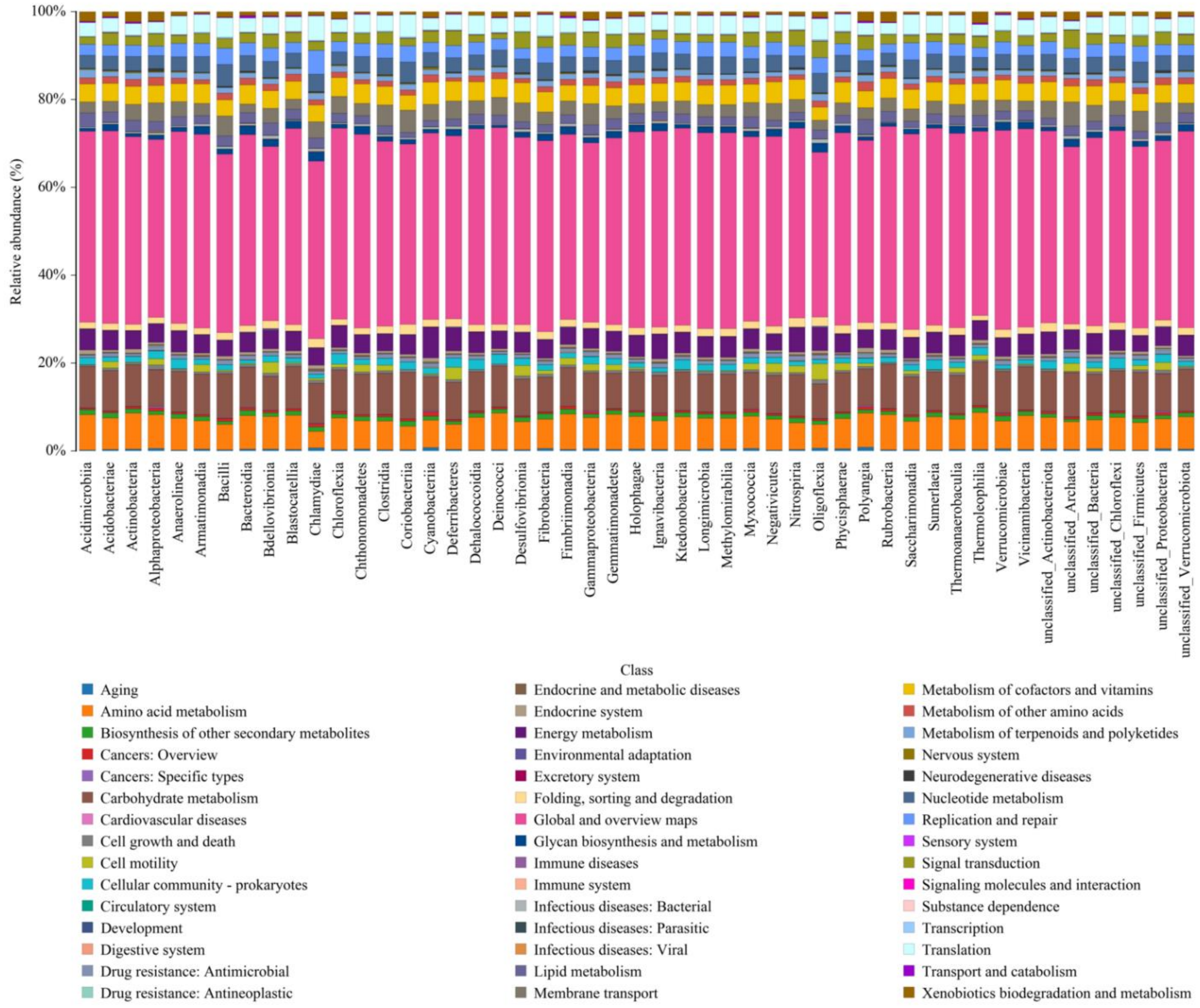

Figure S4. Overview of predicted KEGG pathway composition. Stacked bar plot showing PICRUSt2-predicted KEGG functional categories across bacterial taxa.

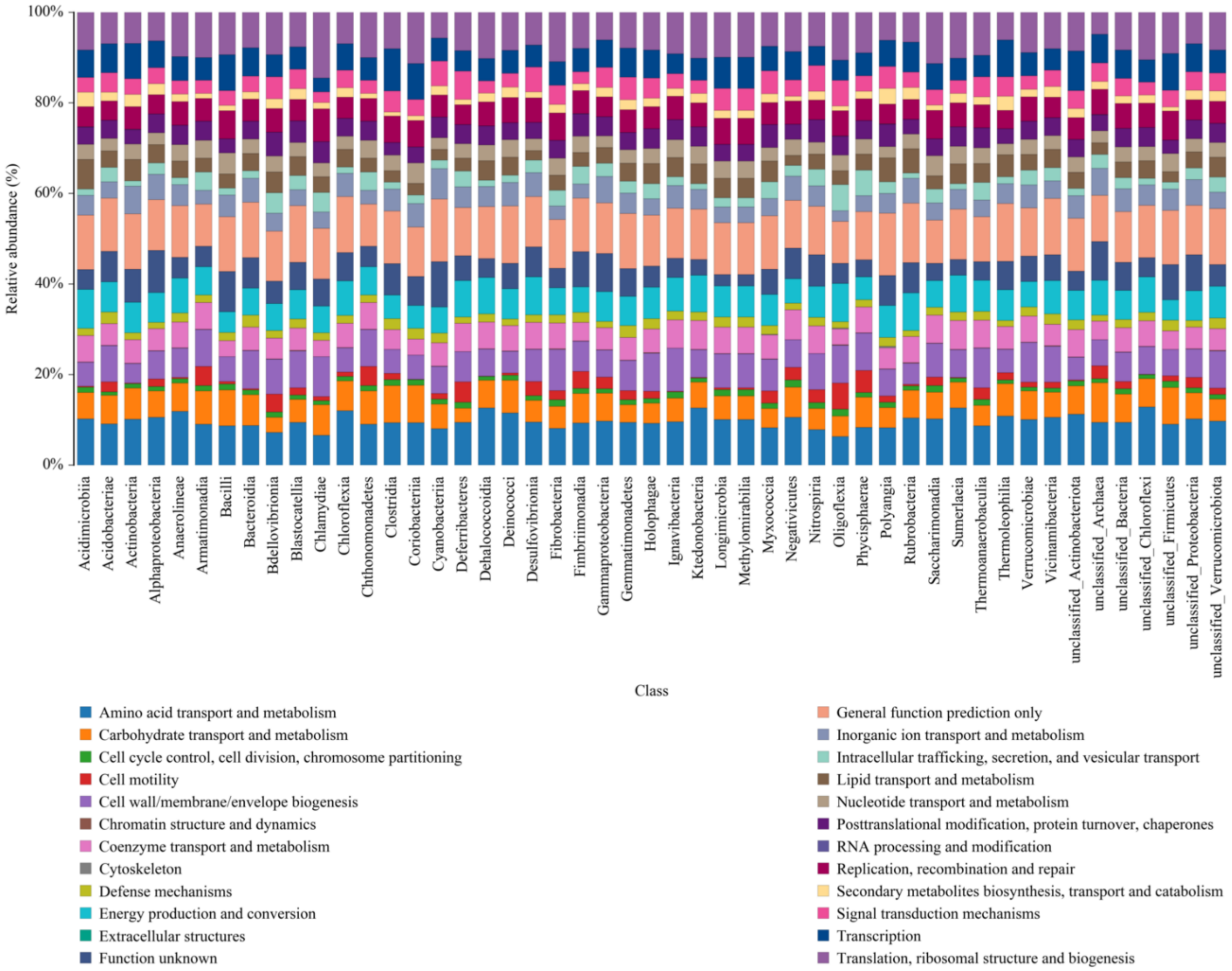

Figure S5. Overview of predicted COG functional composition. Stacked bar plot showing predicted COG functional categories across bacterial taxa based on 16S-derived functional inference.

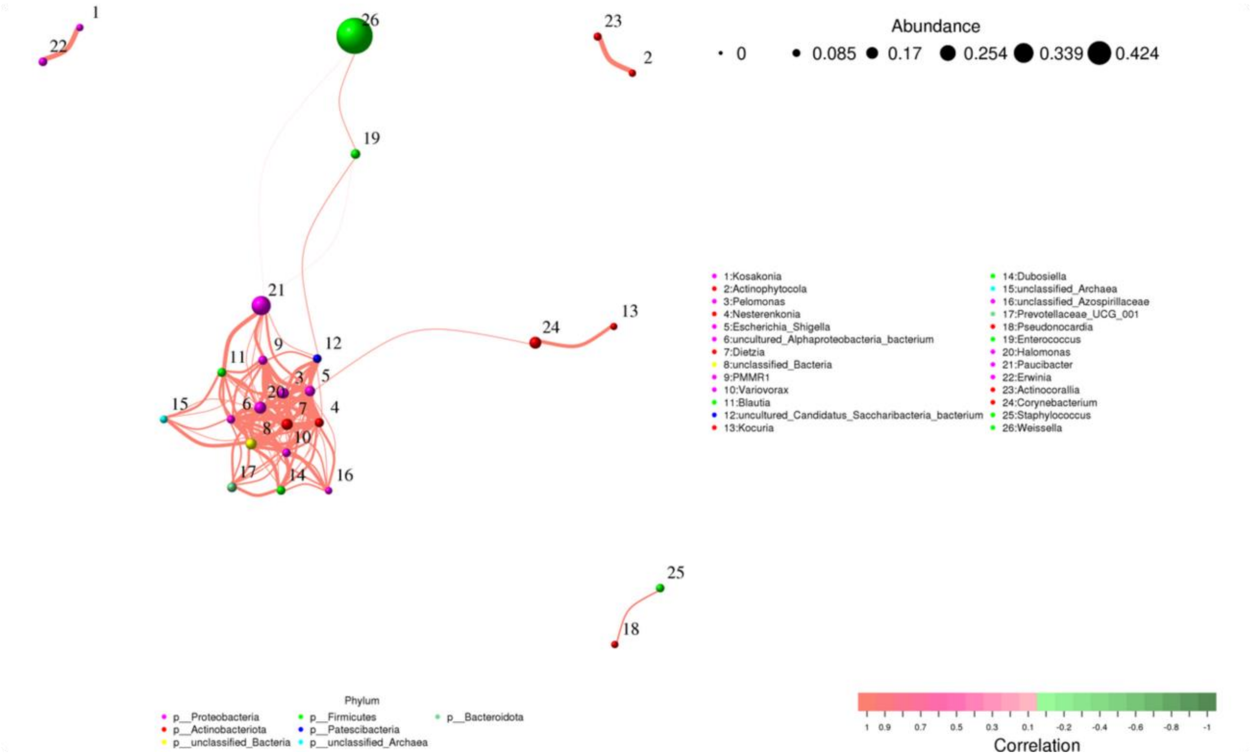

Figure S6. Co-occurrence network of bacterial genera. Spearman correlation-based network showing associations among dominant bacterial genera. Node size represents relative abundance, node color indicates phylum and edge color indicates correlation direction.

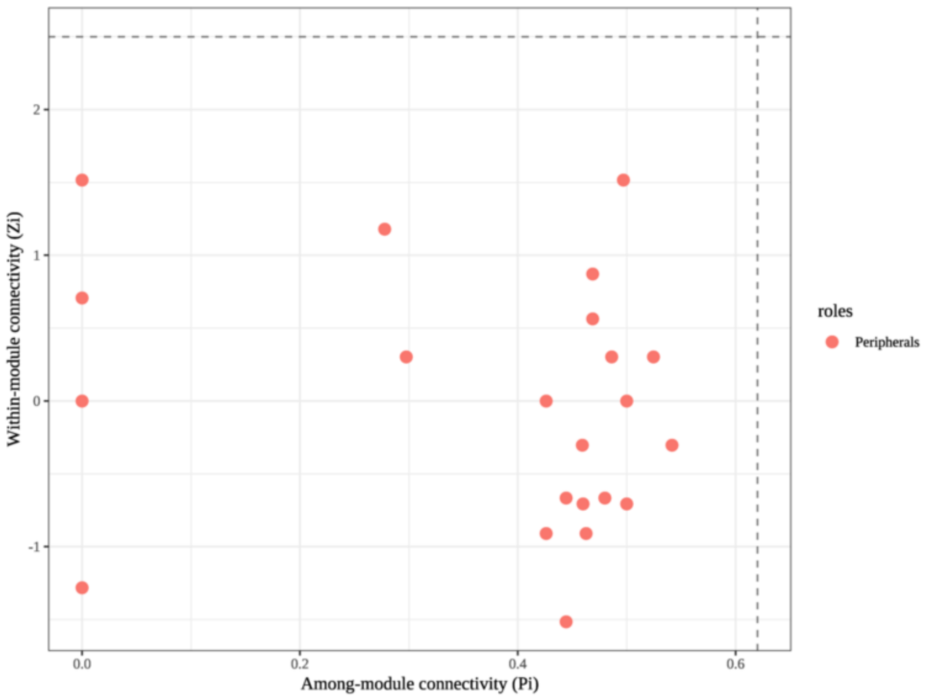

Figure S7. Zi–Pi classification of network nodes. Within-module connectivity and among-module connectivity plot showing the topological roles of bacterial genera in the co-occurrence network.

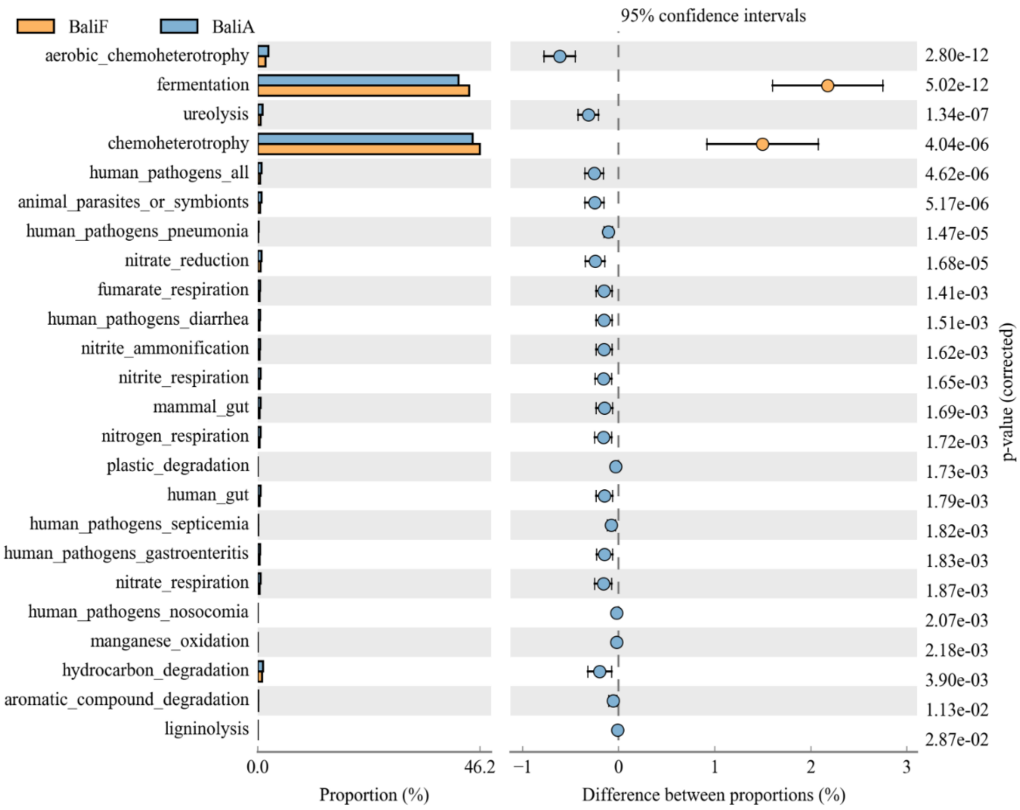

Figure S8. Predicted ecological functions of bacterial communities. FAPROTAX based comparison of predicted ecological functions showing proportional abundance, between sample or between group differences, 95% confidence intervals and corrected significance values for major inferred ecological-functional categories. FAPROTAX results are taxonomy based ecological function predictions and should be interpreted as exploratory, not as direct measurements of microbial activity or metabolism.
